# Contralateral Axillary Lymph Node Metastases at the Time of Primary Breast Cancer Diagnosis: Curative or Palliative Intent?

**DOI:** 10.1155/2013/389013

**Published:** 2013-03-28

**Authors:** C. Zhou, M. C. Richir, M. W. H. Leenders, B. L. A. M. Langenhorst, H. P. Knol, W. H. Schreurs

**Affiliations:** ^1^Department of Surgery, Medical Center Alkmaar, Alkmaar, The Netherlands; ^2^Department of Radiation Oncology, Medical Center Alkmaar, Alkmaar, The Netherlands

## Abstract

Contralateral axillary lymph node metastases (CAMs) in breast cancer patients are uncommon. CAM can be found at the time of primary breast cancer diagnosis or following prior treatment of breast cancer as a recurrence. This distinction may have important implications for disease staging and treatment selection. We report the case of a premenopausal woman with synchronous CAM. Despite extensive multimodality treatment, a recurrence was found 27 months after primary surgery. We reviewed the literature on histopathological tumor characteristics associated with CAM, lymphatic drainage of the breast to other sites than the ipsilateral axilla, and outcome of cases with CAM. This case contradicts current conceptions that CAM only develops from tumors with poor histopathological features. Emerging evidence shows that altered lymphatics play a central role in development of synchronous CAM. It is precisely this etiology that supports the concept that synchronous CAM occurs by lymphatic spread and not by hematogenous spread. Although controversial, treatment of synchronous CAM (without evidence of distant metastases) should therefore be of curative intent.

## 1. Background

Contralateral axillary lymph node metastases (CAMs) in breast cancer patients are uncommon [1]. Traditionally, all cases of CAM have been regarded as distant disease, and as such are treated with systemic therapy, either chemotherapy or hormonal. However, CAM can be found at the time of primary breast cancer diagnosis (synchronous CAM) or following prior treatment of breast cancer as a recurrence (metasynchronous CAM). Such a distinction may reflect different stages of disease and motivate either treatment with curative or palliative intent. 

We report the case of a 47-year-old woman who was found to have synchronous CAM. We aimed to better understand this clinical phenomenon by reviewing the literature on lymphatic drainage of the breast to other sites than the ipsilateral axilla, histopathological tumor characteristics associated with CAM, and treatment and outcome of cases with synchronous CAM.

## 2. Case Report

A 47-year-old premenopausal woman presented to the outpatient breast cancer clinic of our hospital because of a palpable mass in the upper outer quadrant of her left breast. Ultrasonographic examination revealed heterogeneously dense breasts, with a solid mass measuring 3.6 cm × 2.6 cm in her left breast and one smaller solid mass. Axillary ultrasound showed multiple enlarged lymph nodes in the ipsilateral axilla, with a maximum diameter of 7.4 mm. Ultrasound-guided core biopsies of the masses in the left breast and fine-needle aspiration cytology (FNAC) of the lymph node in the left axilla were obtained. Pathological examination of the tissue from each of the solid masses revealed grade III invasive ductal carcinoma. The tumor expressed estrogen receptor and progesterone receptors but did not over-express Her2/Neu. The specimen from the axillary lymph node contained malignant cells. Subsequent whole body positron emission tomography with 18-fluorodeoxyglucose (18FDG-PET) scanning showed positive uptake in both left and right axilla and in the left breast. Consequently, FNAC of right axillary lymph node was obtained, which also contained malignant cells. Computed tomography (CT) of the chest and abdomen after the administration of contrast material and a bone scan showed no evidence of metastatic disease outside of the breast and axillae. 

The patient underwent neoadjuvant chemotherapy consisting of 5-fluorouracil, epirubicin, and cyclophosphamide for six cycles, every three weeks. After extensive discussion, she elected to undergo modified radical mastectomy (MRM) of her left breast, bilateral axillary lymph node dissection, and prophylactic MRM of her right breast. Tissue from the left breast contained a grade III invasive ductal carcinoma 2.0 cm × 2.0 cm, with negative margins. There was no evidence of carcinoma in the tissue from the right breast. Of 5 dissected lymph nodes from the left axilla, 4 contained metastases. Eleven of 14 dissected lymph nodes from the right axilla were positive. Following surgery, she underwent locoregional radiotherapy for a dose of 50 Gy in daily fractions of 2 Gy to the chest wall via opposing tangential fields with 6 MV photons. Overlapping fields were avoided by matched midline technique using the CT simulator. The supraclavicular and internal mammary fossae were not included in the target volume. After discussing the risk and benefits of adjuvant hormonal therapy, she elected to start with adjuvant letrozole. 

Unfortunately, at followup 27 months after primary surgery, a mass was found at the presternum on physical examination. Ultrasound examination revealed a subcutaneous mass measuring 3.3 cm × 1.1 cm. Ultrasound-guided core biopsies of the lesion showed grade III invasive ductal carcinoma that was positive for ER and PR, but negative for Her2/Neu. Subsequent 18FDG-PET/CT showed no evidence of distant metastatic disease.

## 3. Discussion

Contralateral axillary lymph node metastases (CAMs) are uncommonly found at the time of primary breast cancer diagnosis (i.e., synchronous CAM). Incidences of CAM vary between 1.9% and 6% for all breast cancer cases [1–4]. However, studies include mostly cases with CAM as a recurrence (i.e., metachronous CAM). In Devitt's series, only two out of 52 patients had CAM at the time of primary diagnosis [4]. As such, its true incidence is probably much lower than reported in the literature. 

Development of CAM is associated with aggressive histopathological features of the primary breast tumor. Morcos et al. retrospectively analyzed their breast cancer patients with CAM. Twenty-one patients with CAM were compared to 401 breast cancer patients without CAM. They demonstrated that breast cancer patients with CAM had significantly worse histopathological features, such as higher tumor grade (81% grade 3 carcinomas), lymphovascular invasion (LVI) (81%) larger primary breast tumours (95% cT3/cT4 breast carcinoma), ER-receptor negativity (52%), and HER-2 overexpression (42%) [3]. However, most of their patients developed CAM as a recurrence (metachronous CAM), occurring 12–32 months after breast cancer diagnosis. Only 10 of 21 patients had CAM at the time of primary diagnosis. This may have biased their results, as recurrences are more likely to develop from tumors with poor histopathological features. In Huston's series, only one patient out of seven had synchronous CAM. The associated tumor also showed LVI, hormone-receptor negativity and HER-2 overexpression [2]. In comparison, none of these histopathological features were present in our case, which underlines the variability in tumors that give rise to CAM.

In addition to aggressive histopathological features, altered lymphatic spread from the tumor to the contralateral axilla contributes to the development of CAM. Development of alternative routes of lymphatic drainage might be prompted by damage to the usual draining lymphatics. For example, irradiation or previous axillary surgery can lead to this damage [5]. However, alternative lymphatic drainage routes might also be present in patients without previous surgery or radiotherapy. These additional drainage routes have particularly been shown since the introduction of the sentinel lymph node biopsy procedure (SNLP). Although primary drainage of the breast is to the ipsilateral axillary lymph nodes, drainage to other sites such as the supraclavicular and internal mammary nodes can occur in up to 30% of cases [6]. Drainage to the contralateral axilla is obvious when blue dye is seen traversing the subcutaneous lymphatics across the chest wall during SNLP and when the subsequent lymphoscintogram demonstrates a hot spot in the opposite axilla [7, 8]. Haagensen used various tracers to demonstrate another possible route. He hypothesized that tumor cells could spread to the contralateral axillary nodes by permeating through the deep lymphatic plexus of the chest wall [9]. Since in our case no history of previous surgery irradiation was noted and blue dye was not seen traversing over the chest wall during SNLP, it seems reasonable to conclude that the primary breast carcinoma used such an alternative lymphatic drainage route to the contralateral axilla.

The above described studies support that synchronous CAMs develop through lymphatic spread from the primary tumor and not by hematogenous spread. Therefore, synchronous CAM without systemic metastases might be considered as a curative disease because the spread is lymphogenic and not hematogenous. However, this concept remains controversial. For example, no classification for CAM is found in the most recent version of the AJCC Cancer Staging Manual, whereas it used to be classified as distant disease in older versions [10]. Despite of this lack of consensus, patients generally undergo treatment with curative intent.

Only a few reports have included treatment outcomes for synchronous CAM. After a median followup of 27 months, two of Morcos' patients with synchronous CAM were alive without evidence of disease, seven patients were still alive with disease, and one had died, all of whom had both CAM and primary tumor eradicated [3]. Huston et al. describe one patient who is alive without disease after 35 months followup [2]. Our patient was also treated with curative intent but despite extensive multimodality treatment, she developed a recurrence 27 months after primary surgery. Although compelling evidence is lacking, it appears that some patients are curable by eradicating both CAM and the tumor, but prognosis for synchronous CAM is usually poor. 

## 4. Conclusion

Our paper draws attention to the diagnostic and therapeutic challenge posed by the rare phenomenon of synchronous CAM. Although synchronous CAM has been considered as distant disease for several decades, emerging evidence shows that altered lymphatics play a central role in development of synchronous CAM. It is this etiology that supports the concept that synchronous CAM occurs by lymphatic spread and not by hematogenous spread. Although compelling evidence is lacking, treatment of synchronous CAM without evidence of distant metastases should therefore be of curative intent. 

## Abbreviations

CAMs: Contralateral axillary lymph node metastases
FNA: Fine-needle aspiration
18FDG-PET: Positron emission tomography with 18-fluorodeoxyglucose
CT: Computed tomography
MRM: Modified radical mastectomy
SNP: Sentinel lymph node biopsy procedure
LVI: Lymphovascular invasion.
